# 

**Published:** 1842-08-27

**Authors:** 


### CONTENTS OF No. 35.

Dr. I. F. Meigs' Case of Inflammation and Necrosis of the Femur, with Femoral Abscess, terminating fatally on the thirtieth day, 545
ANALECTA: Dr. Haller on the Radical Cure of Reducible Inguiual Hernia, - - - - - - - - - - - - - 547
M. Charriere on Gilding of Surgical Instruments, - - 548
Dr. Peebles Case of Hysterical Affection of the Eyes, with obstinate Closure of the Lids, - - - - - - 548
Dr. Smee's Case of Violent Hysteria in a man, - - - 549
Dr. Roesch on the Nature and Treatment of Scrofula, - - 549
Dr. Thomas Graham Wier's Communication illustrating the durability of the Vaccine Virus, - - - - - - 549
Licentiate Blich's Trial of the Cold Water System, - - 551
Mr. Lyon on Hydrencephalocele, - - - - - - 555
Nitrate of Potass in Hæmoptysis, - - - - - - 556
Mr. Higgins' Case of Traumatic Tetanus, - - - - 556
Ultimate Secreting Structure, - - - - - - - 557
Dr. Duvard's Case of Catalepsy with Transposition of the Senses, - - - - - - - - - - - - 558

